# Topology‐Enabled Finite‐Band Absorption Framework Based on a Bioinspired Nested Dual‐Loop Architecture

**DOI:** 10.1002/advs.77216

**Published:** 2026-08-17

**Authors:** Shaokang Liu, Weicheng Liao, Pinchen Luo, Mengchen Jin, Tangming Yan, Xing Zhao, Qingshan Wang

**Affiliations:** ^1^ College of Mechanical and Electrical Engineering Central South University Changsha People's Republic of China; ^2^ State Key Laboratory of High‐Performance Complex Manufacturing Central South University Changsha People's Republic of China; ^3^ Institute of Light Alloy Central South University Changsha People's Republic of China; ^4^ School of Electronic Science and Engineering University of Electronic Science and Technology of China Chengdu People's Republic of China

**Keywords:** biomimetic structure, finite‐band absorption framework, nested structure, neural network‐genetic algorithm, radar cross section

## Abstract

The Rozanov limit provides a fundamental causality constraint for passive electromagnetic absorbers, but practical ultrabroadband absorbers are also governed by how efficiently structural topology injects, stores, redistributes, and dissipates the available material loss. Here, we propose a topology‐enabled finite‐band absorption framework to evaluate structural loss utilization under fixed material and thickness constraints, rather than replacing the classical Rozanov causality theorem. Inspired by the selective transport mechanism of nuclear pore complexes, a nested dual‐loop architecture is developed to create coupled energy‐transport and dissipation pathways. Combined with Co/Fe3O4 dual‐magnetic composites and neural‐network‐assisted genetic optimization, the absorber achieves continuous ultrabroadband attenuation from 2 to 40 GHz with a thickness of 11 mm. Reference comparisons with planar slab, single‐loop, and pyramid structures show that the dual‐loop topology provides complete band coverage and a finite‐band structural utilization score of 82%. When applied to missile‐scale curved surfaces, the absorber reduces the equivalent radar cross section to 1% of the metallic counterpart. This work establishes a practical topology‐centered evaluation and design route for ultrabroadband, conformal electromagnetic stealth structures.

## Graphical abstract

A nuclear‐pore‐inspired nested dual‐loop architecture redistributes electromagnetic energy through coupled transport, confinement, and dissipation pathways. Integrated with Co/Fe_3_O_4_ dual‐magnetic composites and topology optimization, the 11‐mm‐thick absorber delivers continuous 2–40 GHz attenuation, raises finite‐band structural utilization to 82%, and reduces the equivalent radar cross section to approximately 1% of metal on missile‐scale curved targets.

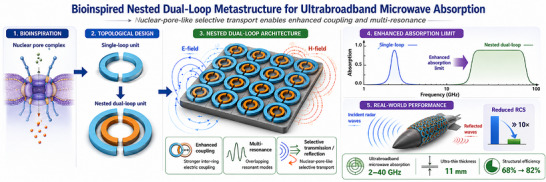

## 1 Introduction

Electromagnetic (EM) absorbers with broad operating bandwidth and reduced thickness are essential for modern stealth platforms, electromagnetic compatibility, and broadband wave‐control systems [1, 2, 3]. However, these two requirements are intrinsically conflicting. Under the constraints of linearity, passivity, and causality, the achievable absorption bandwidth of a finite‐thickness absorber is fundamentally restricted by the Rozanov bound [4], which links thickness to the frequency‐integrated reflection response. As a consequence, thin absorbers often exhibit fragmented absorption bands, insufficient low‐frequency attenuation, and strong dependence on structural thickness [5]. Although substantial progress has been achieved through magnetic–dielectric hybrids, lossy composites, multilayer impedance‐matching strategies, and resonant metamaterials [6, 7, 8], most existing approaches still remain within a material‐centered optimization picture and therefore do not explicitly explain how structural topology contributes to approaching the practical broadband limit [9, 10]. For practical stealth and electromagnetic compatibility systems, the bandwidth‐thickness trade‐off is not a purely academic constraint. Low‐frequency absorption generally requires a long propagation path or magnetic response, while high‐frequency absorption requires impedance continuity and distributed dissipation. Reducing thickness while maintaining continuous low‐to‐high‐frequency attenuation is therefore essential for conformal coatings on aircraft, missiles, unmanned platforms, and compact electromagnetic protection systems.

The classical Rozanov [4] description remains a fundamental causality constraint for passive absorbers and is not replaced in this work. However, for ultrabroadband absorbers spanning multiple octaves, a low‐frequency asymptotic descriptor alone cannot fully distinguish how different structural topologies utilize the same material loss over the target finite band [11]. Higher‐frequency resonances, field redistribution, interference‐assisted confinement, and topology‐regulated propagation pathways all contribute to the measured response [12]. We therefore introduce a finite‐band structural utilization framework to evaluate the practical use of available material loss under fixed thickness and material constraints.

**FIGURE 1:**
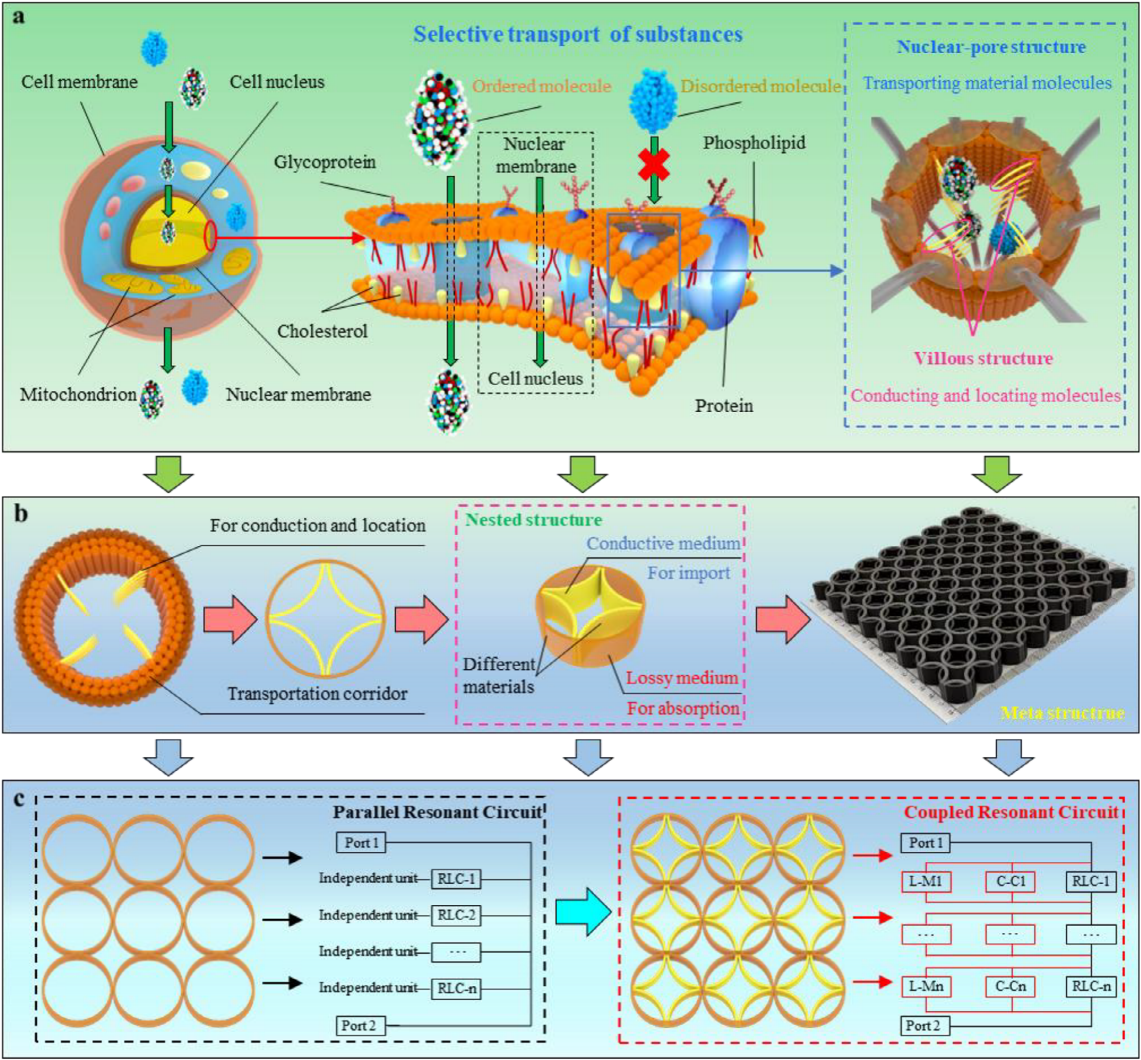
Bioinspired nuclear‐pore‐guided nested dual‐loop design. (a) Nuclear pore structure. (b) Nuclear‐pore‐inspired dual‐loop architecture. (c) Conceptual coupled‐resonator representation used to illustrate topology‐induced energy exchange and resonance redistribution.

Natural transport systems provide an instructive route toward such a topology‐centered design. The nuclear pore complex (NPC), located on the nuclear membrane, realizes selective multi‐channel transport within an extremely compact thickness through nested and hierarchical pathways (Figure 1a). This principle is mapped here to electromagnetic absorption in terms of three physical analogies: selective injection of incident energy into the structure, multi‐channel propagation through nested loops, and distributed dissipation within coupled lossy regions [13]. Thus, the biological prototype is not used as a visual metaphor alone, but as a structural logic for converting isolated loss regions into an interconnected energy‐transport network [14].

Based on this perspective, we propose a topology‐enabled finite‐band absorption framework in which the practical broadband capability of a finite‐thickness absorber is evaluated by structural utilization of material loss. The framework introduces a finite‐band absorption descriptor together with bandwidth expansion, coupling efficiency, and storage‐related descriptors to compare different topologies under the same thickness constraint. Guided by this framework, a bioinspired nested dual‐loop architecture (Figure 1b,c) is developed as its physical realization. By combining dual‐magnetic‐material loss engineering, a conceptual coupled‐resonator representation, and neural‐network‐assisted genetic optimization, the structure converts localized slab dissipation into distributed multi‐loss pathways over a broad spectral range. This theory‐structure synergy enables compact ultrabroadband absorption and pronounced radar‐scattering suppression on curved targets.

## 2 Topology‐Enabled Finite‐Band Absorption Framework

### 2.1 Revisiting the Rozanov Limit

The Rozanov limit provides a fundamental constraint on the bandwidth‐thickness trade‐off of passive absorbers, originating from causality and the Kramers–Kronig relations [15, 16]. In the present work, this limit is used as the physical background rather than as a theorem to be replaced. Our purpose is to define a finite‐band descriptor that can compare how different structural topologies utilize available material loss within a specified operating band.

To retain finite‐band dissipation information, we introduce a broadband absorption descriptor:

(1) $$I=\int_{0}^{\infty }\ln\frac{1}{\left|r\left(s\right)\right|}\;ds$$

where, *r* is the reflection coefficient. This integral quantifies the average finite‐band absorption capability over the selected operating window. It should be regarded as a practical descriptor for topology comparison, not as a new causality bound derived from the Rozanov theorem.

### 2.2 Structural Efficiency and Loss Redistribution

In a realistic absorber, the achievable performance is not governed solely by intrinsic material loss. Structural topology modifies the propagation path, field localization, resonance overlap, and effective utilization of the available material loss. These effects do not create new dissipation channels in a strict material sense, but instead redistribute and amplify the use of existing loss mechanisms across the frequency domain. Accordingly, a topology‐enabled absorption‐capacity reference is expressed as:

(2) $$I_{max}=G_{expand}\;\cdot G_{store}\cdot \eta_{couple}\cdot I_{material}$$

where, *I_material_* represents the intrinsic loss capability of the material system, *G_expand_* characterizes the structural bandwidth expansion factor, *G_store_* accounts for storage‐related field confinement, and *η_couple_* ​quantifies the fraction of incident energy effectively injected into the structure. These quantities are field‐derived and performance‐derived descriptors used for comparing topologies under fixed constraints, rather than independent universal constants.

These factors collectively describe how topology improves the utilization efficiency of material loss by redistributing existing loss channels over frequency and space.

### 2.3 Definition of Structural Design Efficiency

To evaluate broadband absorber performance under fixed thickness, a structural design efficiency is defined as:

(3) $$\eta_{total}=C^{\alpha }\;\eta_{cond}$$

where, *C* denotes the bandwidth coverage ratio under a given reflection threshold, *α* denotes the order, and *η_cond_* represents the finite‐band intrinsic utilization score of the structure within the defined reference set. This score is used for comparative evaluation among structures with the same thickness and material constraints, and does not represent the fraction of a universal physical limit.

### 2.4 Practical Finite‐Band Absorption Envelope

Based on the above framework, the conventional Rozanov background can be connected to a practical finite‐band absorption‐envelope description:

(4) $$B_{eff}=\eta_{total}\;\cdot B_{Rozanov}$$

where, *B_eff_* is the effective achievable bandwidth and *η_total_* reflects the contribution of structural topology within the selected frequency band. Under the assumption of approximately uniform reflection inside the effective absorption band, this relation provides an interpretable bandwidth‐scaling picture. The proposed framework should therefore be understood as a finite‐band practical evaluation framework, rather than a strict replacement of the original causality theorem.

### 2.5 Physical Interpretation

The proposed framework indicates that broadband absorption can be enhanced through three coupled topology‐enabled mechanisms: increased energy injection, broader spectral coverage by nested multi‐order resonances, and improved storage‐and‐dissipation efficiency through field confinement. These mechanisms bridge the gap between material‐intrinsic loss and practical broadband performance while remaining consistent with the passive, causal nature of the absorber.

## 3 Results and Discussion

### 3.1 Material Basis and Multiscale Characterization

**FIGURE 2:**
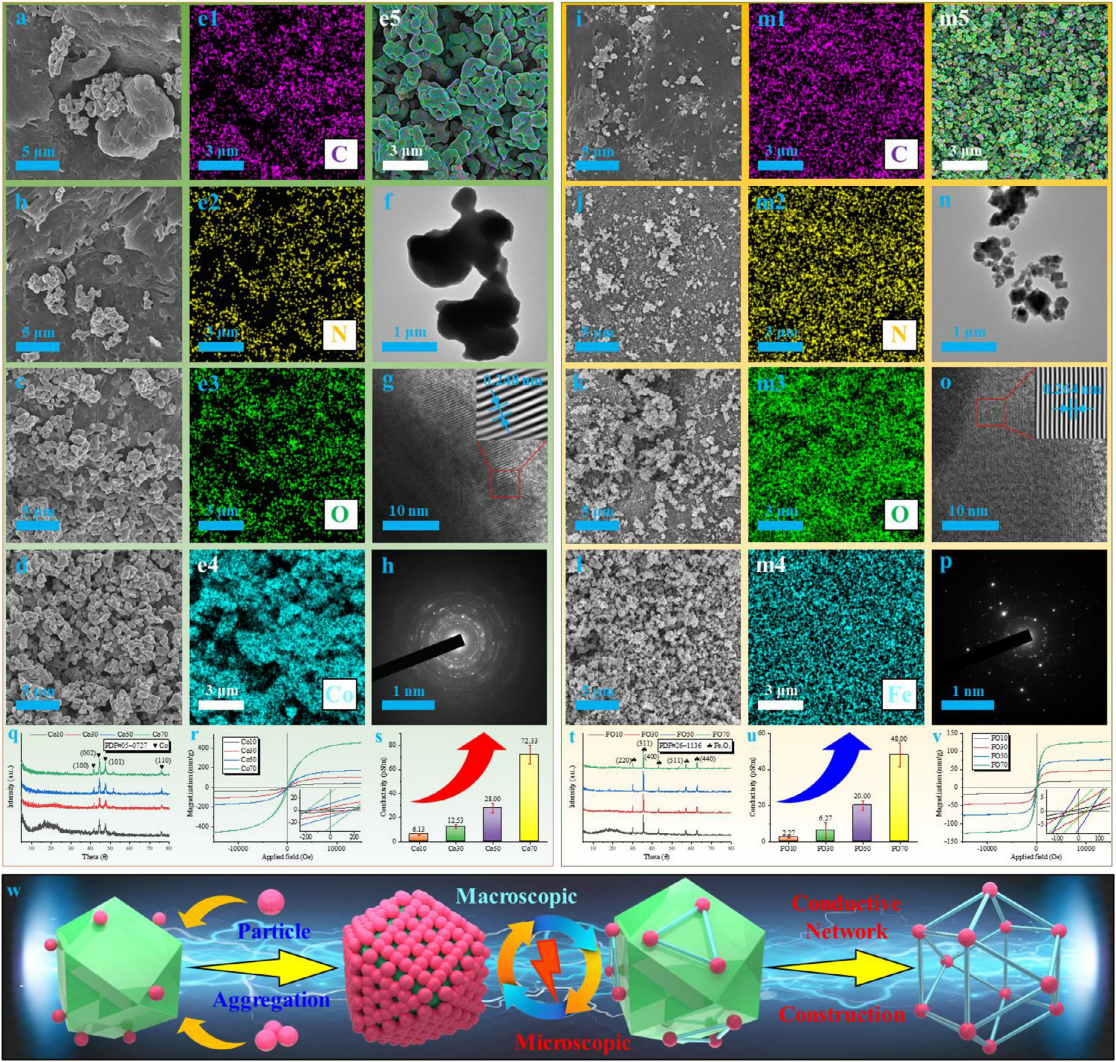
Multiscale structural, magnetic, and conductive characterization of the Co and Fe_3_O_4_ based material systems. SEM of (a) Co10, (b) Co30, (c) Co50, (d) Co70. (e1‐e5) SEM‐mapping of Co70. Co of (f) TEM, (g) HRTEM, (h) SAED. SEM of (i) FO10, (j) FO30, (k) FO50, (l) FO70. (m1‐m5) SEM‐mapping of FO70. FO of (n) TEM, (o) HRTEM, (p) SAED. Co composites of (q) XRD, (r) VSM, (s) conductivity. FO composites of (t) XRD, (u) VSM, (v) conductivity. (w) schematic diagram of electronic channel construction.

Figure 2 establishes the material basis of the proposed absorber by comparing the Co‐based and Fe_3_O_4_‐based filler systems from morphology to macroscopic electromagnetic‐related properties. For the Co series, the SEM images show progressively aggregated metallic particles with irregular block‐like morphology, while the elemental mappings confirm the coexistence of C, N, O, and Co in the composite system. The corresponding TEM, HRTEM, and SAED results indicate that the Co‐containing particles maintain clear crystalline characteristics, consistent with the XRD patterns indexed to metallic Co. In parallel, the magnetic hysteresis loops and conductivity curves reveal that increasing Co content leads to strengthened magnetic response together with a measurable conductive pathway, indicating that the Co‐based phase can simultaneously contribute to magnetic dissipation and current‐assisted loss [17]. The Fe_3_O_4_ series shows a different microstructural route: finer and more uniformly distributed particles are observed in SEM and TEM, while HRTEM and SAED confirm the crystalline Fe_3_O_4_ phase, in agreement with the XRD patterns assigned to the spinel structure [18]. Compared with the Co system, the Fe_3_O_4_‐filled composites provide a distinct magnetic signature and a different conductivity evolution, suggesting complementary electromagnetic roles of the two filler families [19]. The bottom schematic in Figure 2 further summarizes this progression from particle aggregation to macroscopic assembly, microscopic conductive pathways, and finally a cooperative conductive network, implying that the absorber is supported by a dual‐material platform capable of providing both dissipative loss and impedance‐adjustment potential over a broad frequency range. In this sense, Figure 2 is not merely a routine characterization panel; it defines the material baseline on which the topology‐enabled absorption framework can operate.

### 3.2 Intrinsic Electromagnetic Response and Planar Slab Baseline

**FIGURE 3:**
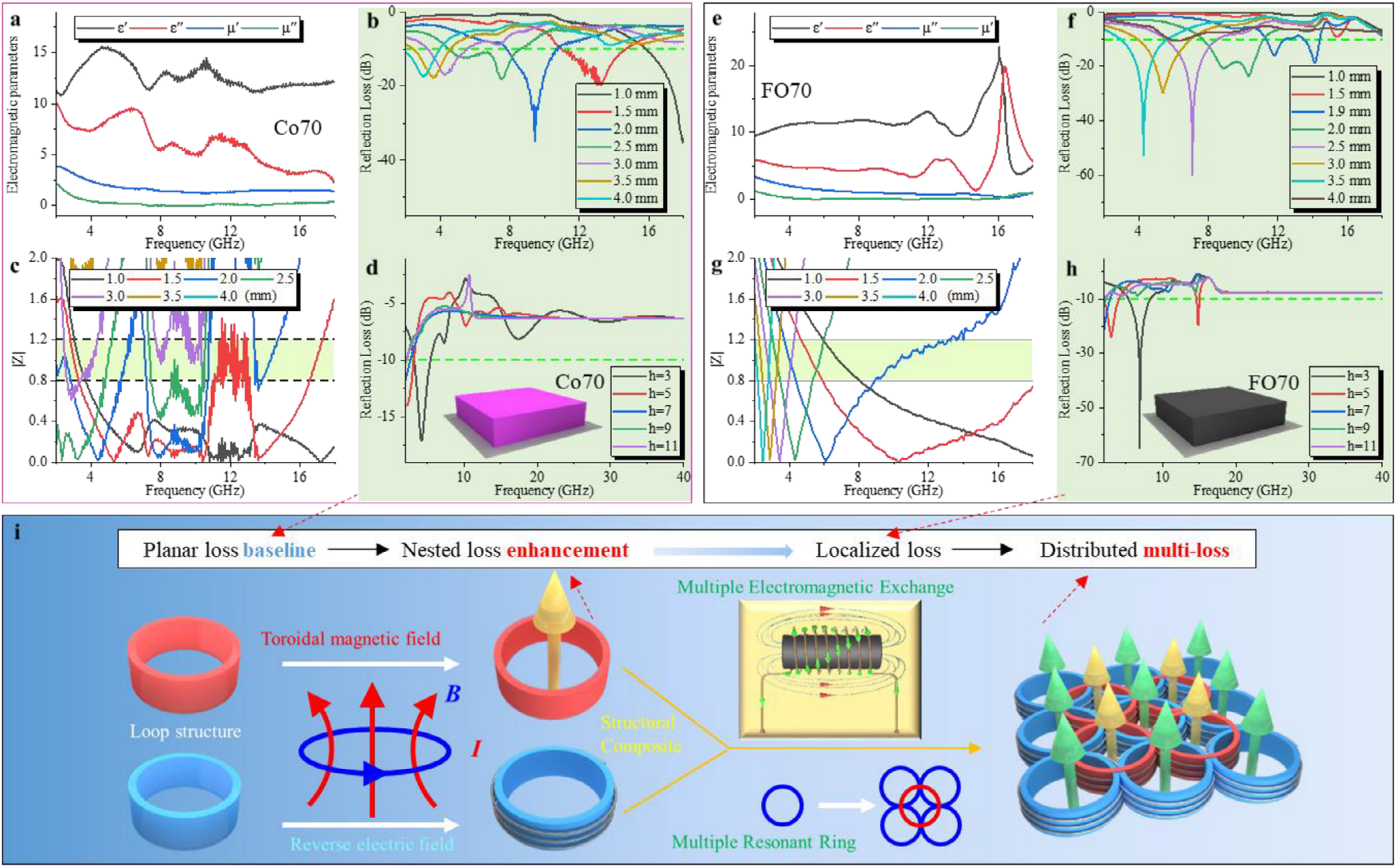
Intrinsic electromagnetic response and planar loss baseline of the optimized Co70 and FO70 systems. The electromagnetic properties of Co70 (a) electromagnetic parameters, (b) RL, (c) impedance |Z| curves. (d) Performance of Co70‐coated flat panel. The electromagnetic properties of FO70 (e) electromagnetic parameters, (f) RL, (g) impedance |Z| curves. (h) Performance of Co70‐coated flat panel. (i) Schematic diagram of topology performance enhancement.

The intrinsic electromagnetic response of the optimized Co70 and FO70 systems is summarized in Figure 3. In the low‐to‐intermediate microwave band, both materials exhibit non‐negligible dielectric and magnetic responses (as shown in Figure 3a–h) [20], as reflected by the frequency‐dependent permittivity and permeability curves. These intrinsic parameters indicate that the dual‐material system already possesses the necessary physical basis for broadband dissipation. However, when these materials are evaluated in planar slab form, the limitation of material‐only optimization becomes evident. The reflection‐loss maps for different slab thicknesses show that both systems can generate deep absorption valleys only in restricted frequency windows. Likewise, the corresponding |Z| curves demonstrate that impedance matching can be approached at selected bands and thicknesses, but such matching remains frequency‐localized rather than continuously maintained over the full target range. This means that the material system itself is strong, but the slab configuration can only utilize that strength in a fragmented manner. The mechanism of Figure 3i makes this point explicit: planar slabs correspond to a “planar loss baseline” dominated by localized single‐loss behavior, whereas nested topology promotes “nested loss enhancement” and gradually converts localized loss into distributed multi‐loss pathways. Therefore, Figure 3 provides the key transition from material capability to structural necessity: intrinsic loss exists, but topology is required to broaden, redistribute, and fully activate it.

### 3.3 Neural‐Network‐Assisted Structural Optimization

**FIGURE 4:**
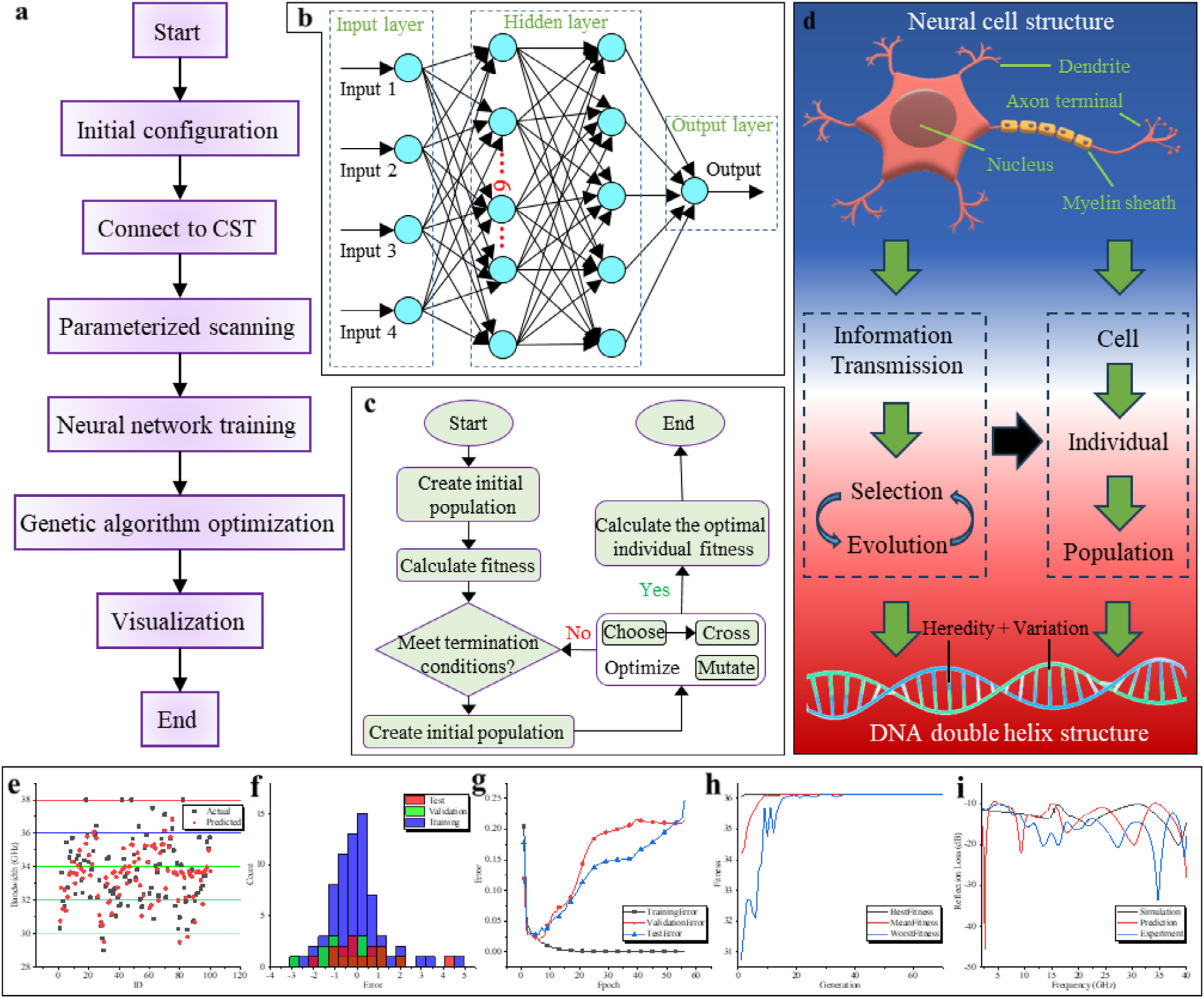
Neural‐network‐assisted genetic optimization of the nested dual‐loop absorber. (a) Optimize the flow chart. (b) Schematic diagram of neural network. (c) Algorithm schematic diagram. (d) Schematic diagram of genetic algorithm. (e) Comparison of training results. (f, g) Error analysis. (h) Schematic diagram of fitting. (i) Comparison of predicted, simulated, and measured performance.

Because the nested dual‐loop architecture introduces a multi‐parameter geometric design space, Figure 4 employs a neural network‐genetic algorithm (NN‐GA) hybrid framework to accelerate structural exploration and identify the optimal parameter set. The geometric input variables include the outer characteristic radius *r1*, two loop/spacing parameters *d1* and *d2*, and the structural height *h*; their candidate values and boundary conditions are summarized in Table S2. The upper panels summarize the workflow, including initial CST‐based parameterization, data acquisition, neural‐network training, and subsequent global search by genetic optimization. The lower panels show the quality of the fitted model through actual‐vs.‐predicted bandwidth comparison, error distribution, training/validation/test loss evolution, GA fitness evolution, and final consistency among simulation, prediction, and experiment. Importantly, the NN‐GA framework is used as a design‐space exploration tool rather than an independent physical model; the absorption mechanism is evaluated by the reflection response, field distribution, impedance behavior, and finite‐band structural utilization metrics.

### 3.4 Coupling‐Enhanced Field Redistribution and Distributed Dissipation

**FIGURE 5:**
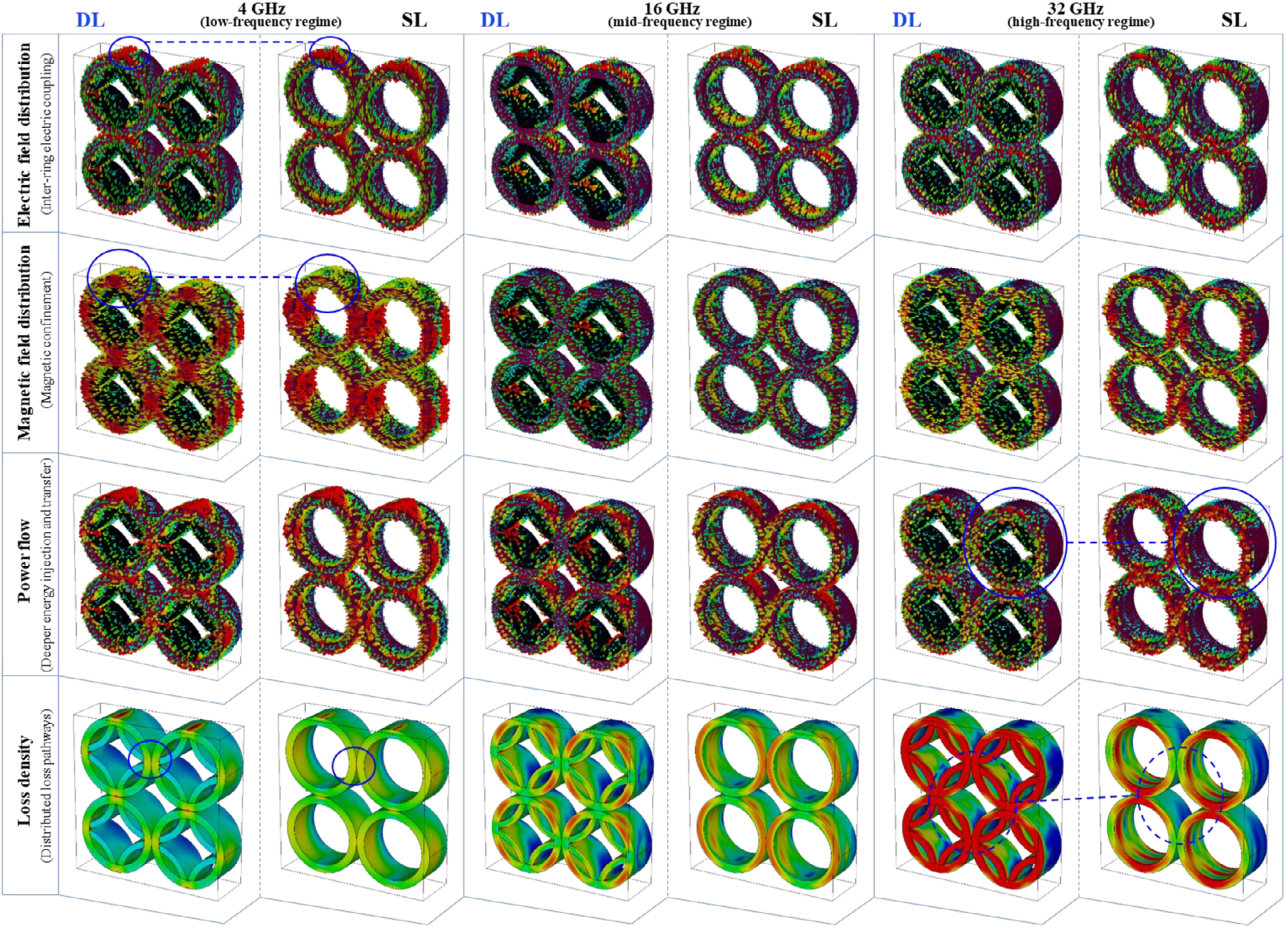
Coupling‐enhanced field redistribution and distributed dissipation in dual‐loop and single‐loop structures.

The physical mechanism underlying the superiority of the nested dual‐loop architecture is directly visualized in Figure 5 through side‐by‐side comparisons between the dual‐loop (DL) and single‐loop (SL) structures at 4, 16, and 32 GHz, corresponding to the low‐, mid‐, and high‐frequency regimes. In the electric‐field maps, the DL configuration exhibits much stronger inter‐ring hotspots, particularly in the interfacial regions between adjacent loops, whereas the SL structure mainly confines the field along individual loop edges. This difference indicates that the nested arrangement activates a coupling‐assisted excitation mode rather than a set of isolated local resonances. The magnetic‐field maps further reinforce this point: DL sustains more continuous and intensified magnetic confinement, especially in the inter‐loop regions, whereas SL shows weaker and more spatially separated magnetic activity. The power‐flow distributions reveal that electromagnetic energy penetrates more deeply and remains more connected in the DL structure, while the SL case is dominated by comparatively shallow and localized power transport. The most decisive contrast appears in the loss‐density maps. At high frequency, the DL structure develops broad, distributed multi‐loss coverage across several coupled regions, while the SL structure still dissipates energy mainly along isolated ring edges. Taken together, these observations establish that the nested dual‐loop topology transforms localized resonance into coupled multi‐mode interaction and reorganizes dissipation over both space and frequency. This directly maps onto the framework introduced in Section 2: enhanced power penetration corresponds to higher *η_couple_*, stronger field confinement supports *G_store_*, and the emergence of multi‐frequency distributed loss pathways gives rise to *G_expand_*. Thus, the nested structure does not introduce a fundamentally new material loss mechanism; rather, it amplifies and redistributes the intrinsic loss provided by the material system, thereby enabling a much more efficient utilization of dissipation across the ultrabroadband regime. The representative frequencies further reveal a progressive spectral evolution rather than an abrupt mechanism switch. At 4 GHz, collective loop excitation and magnetic confinement assist the initial penetration of electromagnetic energy; at 16 GHz, overlapping inter‐loop modes strengthen coupling and resonance overlap; and at 32 GHz, higher‐order edge and interfacial modes form spatially distributed dissipation pathways. This continuous evolution explains how the nested architecture sustains attenuation across the ultrabroadband range.

To further isolate the topology contribution, planar slab, single‐loop, and pyramid reference structures were evaluated under the same thickness condition in the Supporting Information. The nested dual‐loop structure exhibits the highest finite‐band absorption descriptor, complete band coverage, and the highest structural utilization score, confirming that the ultrabroadband response originates from topology‐enabled loss utilization rather than merely from material loss or thickness (Figure S6 and Table S1).

### 3.5 Broadband Absorption and System‐Level Stealth Validation

**FIGURE 6:**
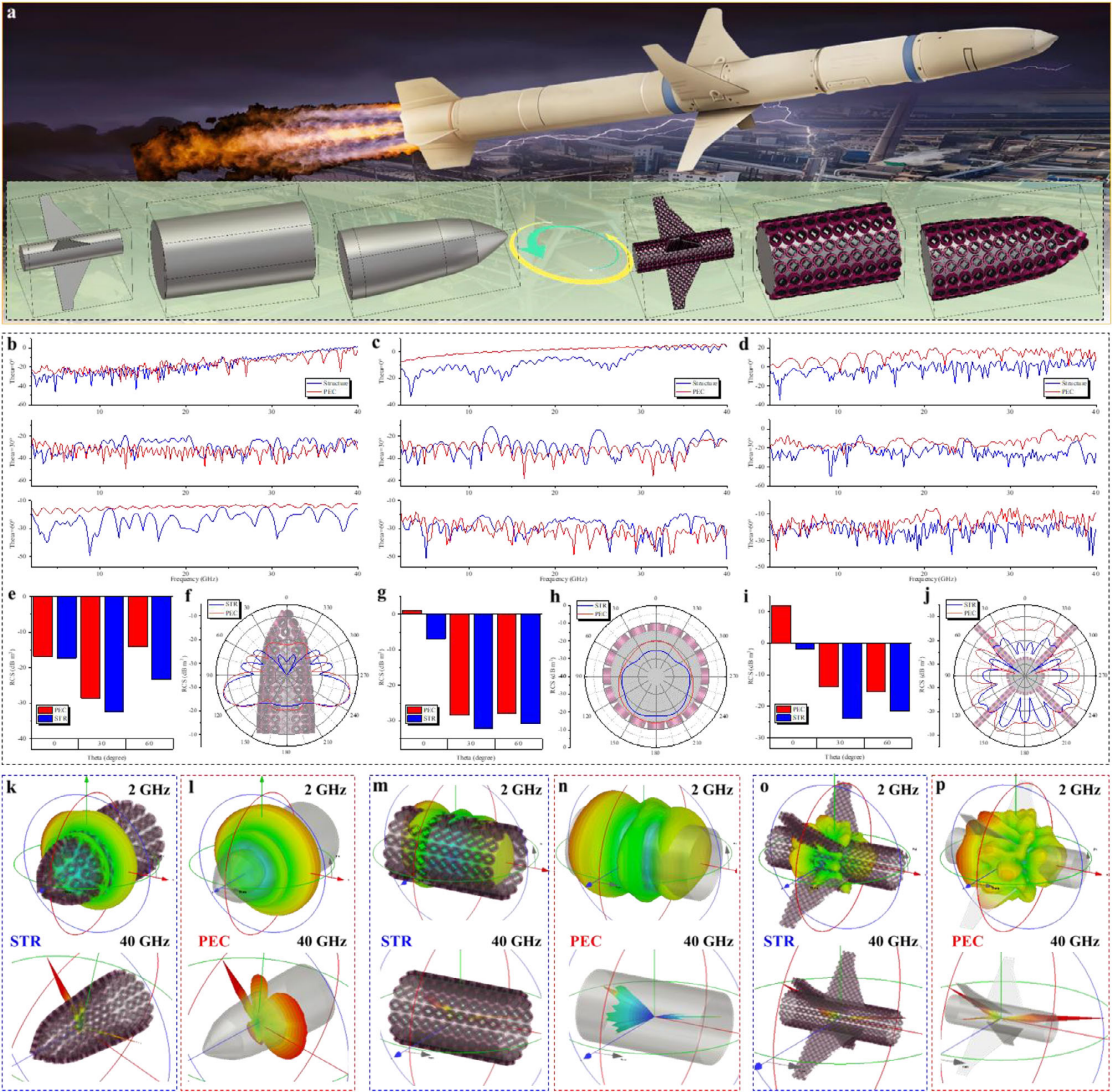
Broadband radar‐scattering suppression of absorber‐coated missile‐scale curved targets. (a) Schematic diagram of split structure coverage. RCS performance with PEC (b,e,f) warhead, (c,g,h) missile body, (d,i,j) missile wing. Comparison of 3D RCS in different frequency (k‐p).

Figure 6 demonstrates that the nested absorber not only performs well at the unit level, but also retains its effectiveness when transferred to realistic curved targets in full‐wave RCS simulations. The upper panel shows representative curved components coated with the proposed absorber; the model is used solely to evaluate conformal radar‐scattering suppression and does not correspond to any specific real platform. The frequency‐domain RCS curves under θ = 0°, 30°, and 60° consistently show lower backscattering for the coated structure than for the PEC reference, indicating that the absorber remains effective over multiple incidence conditions rather than only under ideal normal incidence [21, 22]. The bar charts and polar plots further confirm that the reduction persists when the response is integrated over angular space, while the 3D scattering patterns show substantial contraction of the dominant scattering lobes. Although these RCS results are simulation‐based, they are supported by experimentally measured material parameters, fabricated absorber samples, mechanical/thermal supplementary characterization, and measured unit‐level reflection response.

### 3.6 Mechanism Summary and Topology‐Enabled Finite‐Band Framework

**FIGURE 7:**
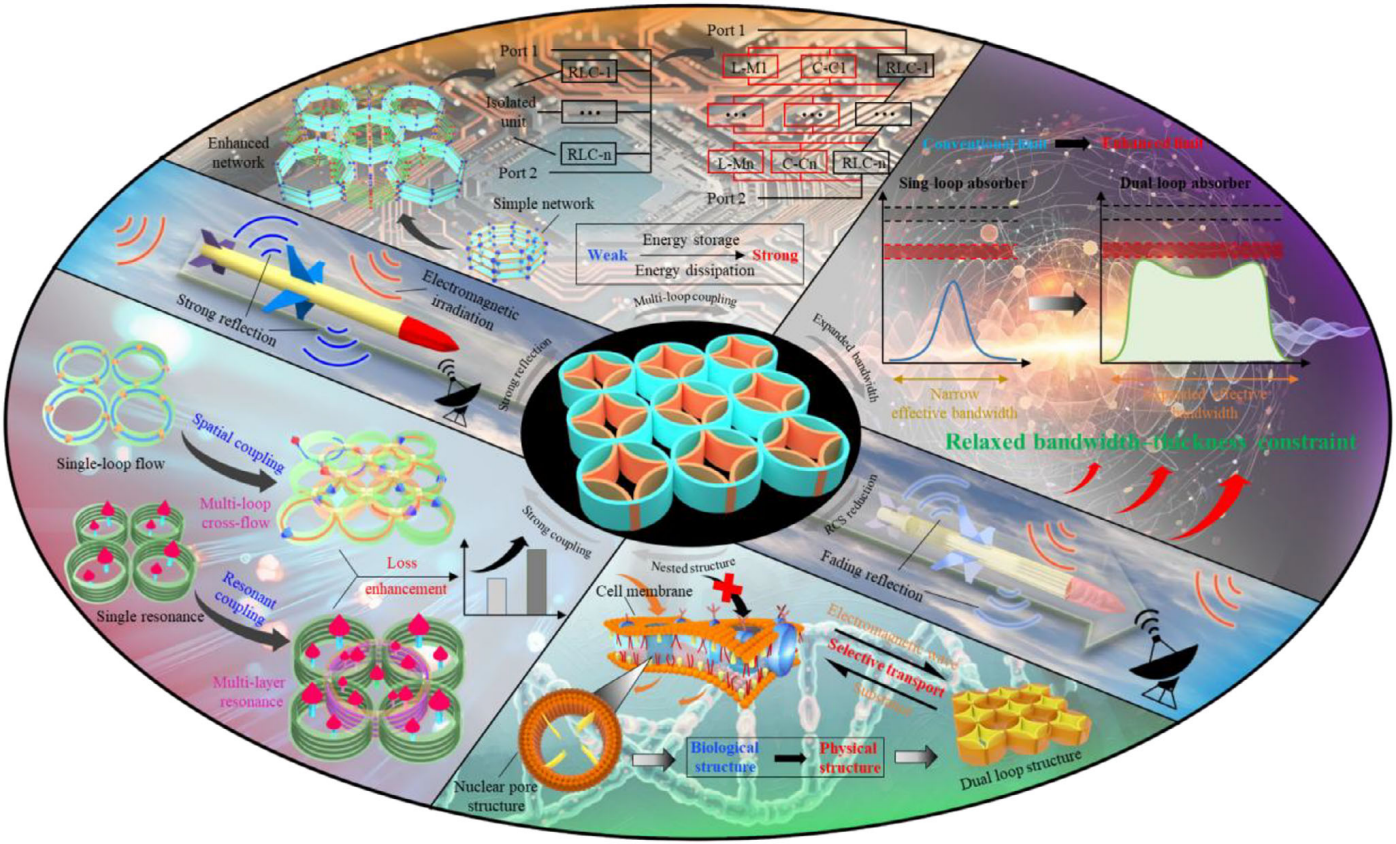
Mechanism summary of the topology‐enabled finite‐band absorption framework.

Figure 7 summarizes the central innovation of this work from the perspectives of biological inspiration, structural topology, electromagnetic coupling, and finite‐band performance evaluation. Unlike conventional absorbers, in which the achievable bandwidth is primarily constrained by intrinsic material loss and weakly coupled resonant pathways, the proposed nested dual‐loop architecture introduces a topology‐enabled redistribution mechanism that reorganizes electromagnetic energy over both space and frequency [23]. This mechanism originates from the nuclear‐pore‐inspired selective transport concept and is translated into a conceptual coupled‐resonator network [24, 25], where isolated resonant branches evolve into interconnected multi‐order channels [26, 27, 28]. As a result, the absorber no longer relies on a single localized loss pathway, but forms a distributed dissipation network capable of sustaining broadband attenuation within a fixed thickness.

From the field perspective, the nested topology simultaneously enhances inter‐ring electric coupling, magnetic confinement, and power penetration, thereby transforming localized resonance into coupled multi‐mode interaction [29, 30, 31]. This effect directly explains the physical meanings of the structural factors introduced in Section 2. Stronger energy injection into the structure contributes to a higher coupling efficiency *η_couple_*, more persistent field confinement supports the storage gain *G_store_*, and the conversion of isolated loss peaks into frequency‐distributed dissipation pathways leads to the bandwidth expansion factor *G_expand_*. In this sense, the structure does not create a fundamentally new material loss mechanism; instead, it amplifies and redistributes the available intrinsic loss so that it can be more fully utilized over an ultrabroad frequency range [32, 33].

This interpretation is fully consistent with the proposed finite‐band structural utilization framework. Under fixed thickness, the practical absorption capability is not determined by material properties alone, but by how effectively the structure expands, stores, and couples electromagnetic energy, as described by the finite‐band absorption‐capacity reference. In the present system, the nested structure exhibits a bandwidth expansion factor of 3.7 and a coupling efficiency of 0.943, ultimately leading to a structural utilization score of 82%. This value is benchmarked against the defined finite‐band reference structure set and should not be interpreted as 82% of a universal fundamental absorption limit.

More importantly, this framework elevates the innovation of the present work from a single high‐performance absorber design to a generalizable topology‐centered design paradigm. The key advance is not only the realization of 2–40 GHz ultrabroadband attenuation at 11 mm thickness, but also the establishment of a structure‐centered performance‐anchoring strategy that links biological hierarchy, coupled energy transport, field redistribution, and system‐level stealth functionality within a unified finite‐band evaluation picture.

## 4 Conclusion

A topology‐enabled finite‐band absorption framework has been proposed for ultrabroadband electromagnetic absorbers by explicitly evaluating structural utilization of material loss under fixed thickness and material constraints. Within this framework, a bioinspired nested dual‐loop architecture was designed to convert localized slab loss into distributed multi‐loss pathways through enhanced coupling, field confinement, and resonance redistribution. Supported by dual‐magnetic‐material loss engineering and neural‐network‐assisted optimization, the resulting absorber achieved ultrabroadband attenuation from 2 to 40 GHz with a thickness of 11 mm, while giving a finite‐band structural utilization score of 82% within the defined reference set. More importantly, the absorber retained strong performance on missile‐scale curved surfaces and reduced the equivalent radar cross section to 1% of the metallic counterpart. These results show that structural topology can act as an independent and quantifiable degree of freedom for improving practical broadband absorption. This work therefore provides both a finite‐band evaluation framework and a scalable design route for wideband, conformal stealth absorbers.

## 5 Experimental Section

### 5.1 Materials

Co and Fe_3_O_4_ powder (2‐3 µm) was acquired from Qinghe Xingrongyuan Metal Materials Co., Ltd. (Hebei, China). PLA was obtained from NatureWorks Inc. (Minnesota, USA), and thermoplastic polyurethanes (TPU) were purchased from Bayer AG (Leverkusen, Germany).

### 5.2 Preparation of Materials and Structures

Initially, PLA powder was placed in an electric constant temperature air blast drying oven and dried at 55°C for 6 h to eliminate moisture. Subsequently, Co, Fe_3_O_4_ and PLA powders were combined with an equal mass of zirconium oxide in a ball mill. The mixture was milled for 4.5 h to produce composite powders; their compositional formulas are detailed in Table S5. The ball‐milled composite powders were then fed into a single‐screw extruder to fabricate composite filaments. Finally, the desired structure was printed using a dual‐nozzle 3D printer.

### 5.3 Characterization and Testing

The morphology and microstructure of the prepared composites were characterized by scanning electron microscopy (SEM) equipped with energy‐dispersive x‐ray spectroscopy (EDS) for elemental mapping, as well as transmission electron microscopy (TEM), high‐resolution TEM (HRTEM), and selected‐area electron diffraction (SAED). The crystalline phases were identified by x‐ray diffraction (XRD). The magnetic properties were measured using a vibrating sample magnetometer (VSM), and the electrical conductivity of the representative samples was determined at room temperature. These measurements were used to establish the structural, magnetic, and conductive basis of the absorber system.

The complex permittivity and permeability of the optimized composite materials were measured using a vector network analyzer (VNA) combined with a coaxial‐line method over the target frequency range. Based on the measured scattering parameters, the electromagnetic parameters were extracted through a standard transmission/reflection inversion procedure. The reflection loss (RL) of planar slabs with different thicknesses was then calculated according to transmission‐line theory, and the normalized input impedance was used to evaluate the impedance‐matching behavior of the material systems.

The electromagnetic response of the nested dual‐loop structure was analyzed using CST Studio Suite. Unit‐cell simulations were carried out under plane‐wave excitation, and the measured electromagnetic parameters of the optimized Co70 and FO70 systems were imported into the corresponding lossy regions. Electric‐field, magnetic‐field, power‐flow, and loss‐density distributions were extracted to investigate the coupling‐enhanced dissipation mechanism. For system‐level validation, missile‐scale curved targets coated with the absorber were further modeled, and the radar cross section (RCS) was calculated under different incident angles and frequency ranges, with bare perfect electric conductor (PEC) targets used as references. The simulated reflection response was finally combined with the absorption‐integral framework proposed in this work to evaluate the topology‐enabled practical bound and the structural design efficiency.

For all finite‐band descriptors reported in this work, the reflection‐loss data were interpolated onto a common frequency grid. The threshold‐crossing points for effective absorption bandwidth were determined by linear interpolation, and the finite‐band absorption descriptor was calculated from the capped integral of ln(1/|S11|) over the target frequency band to avoid over‐weighting isolated extremely deep absorption peaks.

## Author Contributions

The work described in this article is the collaborative development of all authors. S.L. conceived the idea. S.L. carried out the theoretical, analytical, and numerical analysis. W.L., P.L., and M.J. was responsible for designing, fabricating, and measuring the proof‐of‐concept sample. S.L. and T.Y. contributed to different aspects of the theoretical analysis. S.L contributed to writing the manuscript. X.Z. and Q.W. supervised the work.

## Funding

The authors gratefully acknowledge the financial support from the National Natural Science Foundation of China (Grant no. 51705537, 52075554 and 52475145), the Natural Science Foundation of the Hunan Province of China (Grant No. 2022JJ20070 and 2021JJ30841), the Science and Technology Innovation Program of Hunan Province (No. 2023RC3029) and Central South University Innovation‐Driven Research Programme, China (Grant No. 2023CXQD049).

## Conflicts of Interest

There are no conflicts of interest.

## Supporting information




**Supporting File**: advs77216‐sup‐0001‐SuppMat.docx.

## Data Availability

The data can be obtained from the corresponding author based on the requirements.
