# Supplementary material for: Topology‐Enabled Finite‐Band Absorption Framework Based on a Bioinspired Nested Dual‐Loop Architecture
Source: Adv Sci (Weinh). 2026 Aug 17:e77216. Online ahead of print. doi: 10.1002/advs.77216 (PMC13479594; doi:10.1002/advs.77216)
Supplement: Supplementary file 1 [file ADVS-9999-e77216-s001.docx]

Topology-Enabled Finite-Band Absorption Framework Based on a Bioinspired Nested Dual-Loop Architecture

*Shaokang Liu^1,2^, Weicheng Liao^1,2^, Pinchen Luo^1,2^, Mengchen Jin^3^, Tangming Yan^4^, Xing Zhao^3^, Qingshan Wang^1,2,^ ^*^*

1 College of Mechanical and Electrical Engineering, Central South University, Changsha, 410083, P.R. China

2 State Key Laboratory of High-Performance Complex Manufacturing, Central South University, Changsha, 410083, P.R. China

3 Institute of Light Alloy, Central South University, Changsha, 410083, P.R. China

4 School of Electronic Science and Engineering, University of Electronic Science and Technology of China, Chengdu, 611731, P.R. China

E-mail(s): qingshanwang@csu.edu.cn

# 1 Theoretical supplement

The Rozanov limit, originally proposed by Konstantin N. Rozanov, provides a fundamental causality constraint linking absorber thickness, bandwidth, and reflection for passive linear absorbing layers. Its theoretical basis originates from the Kramers-Kronig (K-K) relations, which require the complex permittivity and permeability of any passive medium to satisfy causality. Accordingly, the real part of the permittivity can be written as

$\epsilon^{'}=1+\frac{2}{\pi}P\int_{0}^{\infty} \frac{\omega'\epsilon"(\omega')}{{\omega'}^{2}-\omega^{2}}d\omega'$

(s1)

where ε′(ω) is the real part of the permittivity and ω is the angular frequency. To further describe the spectral response in the complex plane, the Hilbert-transform form of the K-K relation can be applied to the reflection coefficient, giving

$\arg r(\omega)=-\frac{2\omega}{\pi}P\int_{0}^{\infty} \frac{ln\left| r(\omega') \right|}{{\omega'}^{2}-\omega^{2}}d\omega'$

(s2)

which can be simplified to

$\int_{0}^{\infty} \frac{ln\left| 1/r(\omega) \right|}{\omega^{2}}d\omega=\frac{\pi}{2}\tau$

(s3)

where τ is the low-frequency group delay. Combined with the Bode-Fano matching limit, this relation indicates that perfect matching over a finite bandwidth cannot be achieved for an arbitrary passive network. Rozanov extended this principle to absorbing layers and obtained the following integral constraint for the reflection spectrum of a passive time-invariant absorber under normal incidence in free space

$\int_{0}^{\infty} \frac{\ln(1/\left| r(\omega) \right|}{\omega^{2}}d\omega\leq\frac{\pi d}{c}(\sqrt{\mu_{s}\varepsilon_{s}}-1)$

(s4)

where *d* is the absorber thickness, *c* is the speed of light in vacuum, and *μ_s_* and *ε_s_* are the static permeability and permittivity, respectively. This expression defines the classical theoretical boundary of the Rozanov limit. If an idealized full-band absorption target of below –10 dB from 2 to 40 GHz is substituted into this framework, one obtains

$d_{min}\approx\frac{8.31}{\sqrt{\mu_{s}\varepsilon_{s}}-1}$

(s5)

For magneto-dielectric composite absorbers, *μ_s_ε_s_* is typically in the range of 16–64, which leads to *d_min_*≈2.8 mm when an idealized full-band -10 dB target from 2 to 40 GHz is substituted into the simplified static-parameter picture. Such a result should not be interpreted as a directly achievable design thickness for practical ultrabroadband absorbers. Rather, it shows that the low-frequency group-delay representation compresses spectral information into a single asymptotic descriptor and does not by itself distinguish how different finite-band topologies utilize medium- and high-frequency loss channels. Therefore, the following analysis introduces finite-band descriptors for comparative structural evaluation, while the original Rozanov limit remains the underlying causality constraint.

To retain broadband dissipation information, the absorber is recast as a single-port reflection system, and the reflection coefficient is expressed in the Laplace domain as

$r\left( s \right)=D+C{(sI-A)}^{-1}B$

(s6)

where *A*∈**ℝ**^N×N^, *B*∈**ℝ**^N×1^, *C*∈**ℝ**^1×1^, *D*∈**ℝ** or **ℂ**. The low-frequency group delay is

$\tau=\frac{d}{d\omega}argr\left( j\omega\right)|_{\omega\to0}$

(s7)

and can be rewritten as

$\frac{d}{d\omega}argr\left( j\omega\right)\mathfrak{=I}(\frac{1}{r\left( j\omega\right)}\frac{dr\left( j\omega\right)}{d\omega})$

(s8)

By differentiating Eq. s6

$\frac{dr}{\mathrm{ds}}=-C{(sI-A)}^{-2}B$

(s9)

and along *s*=*jω*

$\frac{dr(j\omega)}{d\omega}=j\frac{dr}{\mathrm{ds}}|_{s=j\omega}$

(s10)

which yields

$\tau\mathfrak{=I}\left( \frac{jr_{s}\left( 0 \right)}{r\left( 0 \right)} \right), r_{s}\left( 0 \right)\equiv\frac{dr}{\mathrm{ds}}|_{s=0}=-C\left( -A \right)^{-2}B=-CA^{-2}B$

(s11)

$\tau\mathfrak{=I}\left( \frac{-jCA^{-2}B}{r\left( 0 \right)} \right)$

(s12)

with

$r\left( 0 \right)=D+C{(0I-A)}^{-1}B=D-CA^{-1}B$

(s13)

Using rational fitting in the impedance domain, the present absorber gives r(0)= (0.994221+0.008093j), corresponding to τ= 0.309928. Since the Rozanov limit primarily constrains the extremely low-frequency regime, a dimensionless broadband absorption integral is introduced here:

$I=\int_{0}^{\infty} \ln\frac{1}{\left| r(s) \right|}ds$

(s14)

For the present system, the calculated finite-band descriptor values are *I_actual_*= 2.187, *I_slab_*=0.988, *I_pyramid_*=2.133. In contrast to Eq. s3, the low-frequency weighting factor 1/*ω*^2^ is not used here; instead, the descriptor characterizes average broadband absorption capability within the selected frequency window. This quantity is introduced for finite-band topology comparison and should not be regarded as a new fundamental causality integral. The effective response is jointly determined by intrinsic material loss, thickness/interference loss, topology-enabled field redistribution, impedance matching, leakage/scattering, and storage distribution. In this framework, the material contribution is intrinsic and transferable, whereas the structural contribution does not create a fundamentally new dissipation mechanism; instead, it redistributes and activates the originally bandwidth-limited material loss over frequency through changes in field distribution and propagation pathways.

Accordingly, a topology-enabled absorption-capacity reference is written as

$I_{max}=G_{expand}\cdot G_{store}\cdot\eta_{couple}\cdot I_{material}$

(s15)

where *I_material_* is the best dimensionless intrinsic loss integral of the material system under the selected finite-frequency and physical constraints. In the present case, *I_material_*=0.627. This expression is used as a phenomenological reference for comparing the utilization of material loss by different topologies; it is not claimed to be a universal maximum imposed by causality. The structural bandwidth-expansion factor is defined as

$G_{expand}=\frac{\left| \Delta f_{struct} \right|}{\left| \Delta f_{mat} \right|}$

(s16)

with

$\Delta f_{mat}=\left\{ f\in\left[ f_{1},f_{2} \right]:\left| r_{mat}\left( f \right) \right|\leq R_{0} \right\}$

(s17)

$\Delta f_{struct}=\left\{ f\in\left[ f_{1},f_{2} \right]:\left| r_{sturct}\left( f \right) \right|\leq R_{0} \right\}$

(s18)

Here, ∆*f_mat_* denotes the optimal effective absorption bandwidth of the planar material reference under the same thickness, whereas ∆*f_struct_* denotes that of the designed structure. For the present system, ∆*f_mat_* = 10.26 GHz and ∆*f_struct_* = 38 GHz, giving *G_expand_* = 3.7. The factor therefore quantifies finite-band bandwidth expansion relative to a defined reference rather than an absolute physical bound.

Here, ∆*f_mat_* denotes the optimal effective absorption bandwidth of the single-layer planar slab under the same thickness, whereas ∆*f_struct_* denotes that of the designed structure. For the present system, ∆*f_mat_* =10.26 and ∆*f_struct_* =38, giving *G_expand_* =3.7.

The coupling efficiency is further defined as

$\eta_{couple}=\frac{\int_{f_{1}}^{f_{2}} \omega\left( f \right)\eta_{couple}\left( f \right)df}{\int_{f_{1}}^{f_{2}} \omega\left( f \right)df}, \omega\left( f \right)=\ln\frac{1}{\left| r(f) \right|}$

(s19)

which measures the fraction of incident energy effectively injected into the structure and hence available for dissipation. The calculated value is *η_couple_* = 0.943. To avoid overinterpretation, ηcouple and *G_store_* are treated as field-derived mechanistic descriptors extracted under identical excitation and normalization conditions. They are used to compare energy injection and storage behavior among reference structures, rather than as independent material constants. To characterize the ability of the structure to retain and dissipate the injected energy, the storage gain is introduced as

$G_{store}=\frac{\int_{f_{1}}^{f_{2}} \omega\left( f \right)P_{loss}^{struct}\left( f \right)df}{\int_{f_{1}}^{f_{2}} \omega\left( f \right)P_{loss}^{slab}\left( f \right)df}$

(s20)

Because the energy entering the structure is not dissipated instantaneously, effective broadband absorption requires sustained storage-and-release capability during the dissipation process. Finally, the finite-band structural utilization score is defined as

$\eta_{total}=C^{\alpha}\eta_{cond}$

(s21)

$C=\frac{{EAB}_{-10dB}}{f_{2}{-f}_{1}}$

(s22)

$\eta_{cond}=\frac{I_{B}^{cap}}{I_{max}}$

(s23)

where *η_cond_* is the intrinsic finite-band utilization score, *C* is the band-coverage ratio under the -10 dB criterion, and α is the coverage order. To emphasize continuous broadband performance, *α* = 2 is adopted here. The score is defined within the present fixed-thickness, fixed-frequency-window, and reference-structure framework. It is therefore a comparative topology metric, not the fraction of a universal absorption limit. Under this definition, the nested dual-loop structure reaches a structural utilization score of *η_struct_* = 82% for the 38 GHz effective bandwidth. Reference structures were recalculated using the same finite-band formula and are summarized in Table S1.

# 2 Characterization and testing supplement

Figure S1–S4 provide supplementary evidence for the chemical composition, interfacial bonding, mechanical robustness, and thermal transport capability of the representative material systems. Specifically, Figure S1 presents the XPS spectra of Co70 and FO70, confirming their elemental composition and surface chemical states. Figure S2 shows the FTIR spectra, verifying the coexistence of the polymeric matrix and inorganic magnetic fillers. Figure S3 summarizes the mechanical properties and impact resistance, indicating that the printed composites retain sufficient structural robustness. Figure S4 presents the thermal conductivity of the Co- and FO-series samples, showing that the heat-transport capability increases markedly with filler loading.

**Figure S1** XPS spectra of the representative composites. **a-d)** XPS spectra of Co70, including the survey spectrum and the high-resolution Co 2p, C 1s, and O 1s spectra. **e-h)** XPS spectra of FO70, including the survey spectrum and the high-resolution Fe 2p, C 1s, and O 1s spectra.

The results confirm the elemental composition and surface chemical states of the representative Co- and Fe₃O₄-based systems.

**Figure S2** FTIR spectra of the representative composite systems. **a)** FTIR spectra of the Co-series samples. **b)** FTIR spectra of the FO-series samples.

The characteristic absorption bands verify the coexistence of the polymeric matrix and the inorganic magnetic fillers in the composite systems.

**Figure S3** Mechanical and impact-response properties of the representative composites. **a)** Mechanical properties of the selected samples. **b)** Impact-resistance curves showing the force-time and energy-time responses under dynamic loading.

The results indicate that the printed composites maintain sufficient mechanical robustness for structural fabrication and practical deployment.

**Figure S4** Thermal conductivity of the composite systems. **a)** Thermal conductivity of the Co-series samples. **b)** Thermal conductivity of the FO-series samples.

The thermal conductivity increases progressively with filler loading, indicating the formation of more effective heat-transfer pathways in the highly filled composites.

**Figure S5** Comprehensive electromagnetic parameters, planar reflection-loss behavior, and impedance-matching characteristics of the Co- and FO-series composites. **a1–a3)** Dielectric constant (*ε′*), corresponding planar reflection loss (RL), and normalized impedance |*Z_in_*/*Z_0_*| of the Co-series samples. **b1–b3)** Dielectric loss factor (*ε′′*), corresponding RL, and |*Z_in_*/*Z_0_*| of the Co-series samples. **c1–c3)** Real permeability (*μ′*), corresponding RL, and |*Z_in_*/*Z_0_*| of the Co-series samples. **d1–d3)** Imaginary permeability (*μ′′*), corresponding RL, and |*Z_in_*/*Z_0_*|of the Co-series samples. **e1–e3)** Dielectric constant (*ε′*), corresponding planar reflection loss (RL), and normalized impedance |*Z_in_*/*Z_0_*| of the FO-series samples. **f1–f3)** Dielectric loss factor (*ε′′*), corresponding RL, and |*Z_in_*/*Z_0_*| of the FO-series samples. **g1–g3)** Real permeability (*μ′*), corresponding RL, and |*Z_in_*/*Z_0_*| of the FO-series samples. **h1–h3)** Imaginary permeability (*μ′′)*, corresponding RL, and |*Z_in_*/*Z_0_*| of the FO-series samples.

These results provide the full planar electromagnetic baseline for screening the representative Co70 and FO70 systems used in the main-text analysis.

# 3 Effective impedance and magnetic-loss interpretation

For a metal-backed one-port absorber, transmission is negligible and the effective normalized input impedance can be retrieved from the same complex reflection coefficient as Z_in_ = (1+S_11_)/(1- S_11_). A separately plotted Z_in_ curve is therefore a one-to-one algebraic transformation of the measured or simulated complex S11 rather than an independent mechanism measurement. Moreover, for the present strongly heterogeneous three-dimensional dual-material architecture, a single retrieved port impedance is necessarily a homogenized scalar quantity and cannot resolve the local inter-loop energy exchange. The material-level impedance-matching baselines are already provided in Figure 3 and Figure S5, whereas the complete structure is evaluated through its reflection response, electric and magnetic fields, loss density, and power-flow distributions.

The magnetic contribution is evaluated from the measured permeability spectra and the associated magnetic-loss descriptors. The magnetic loss tangent is defined as tan δ_μ_ = μ″/μ′, while the local time-averaged magnetic dissipation density is

$p_{m}=\frac{\omega\mu_{0}\mu"\left| H \right|^{2}}{2}$

(s24)

These quantities provide quantitative material- and field-level measures of the available magnetic dissipation. However, dielectric, conductive, magnetic, interfacial, and impedance-matching effects are coupled through the same Maxwell solution; therefore, a unique additive percentage of magnetic loss cannot be inferred from the total reflection-loss curve without imposing an artificial decomposition. Figure S5 quantifies the material-level magnetic response, while Figures S9-S12 show how the nested architecture spatially activates and redistributes it.

# 4 Topology-dependent reflection-loss comparison

**Figure S6** Topology-dependent reflection-loss comparison of different reference structures. **a)** Simulated and experimental reflection-loss curves of the nested dual-loop (DL) structure. **b)** Simulated reflection-loss curves of the single-loop Co- and FO-based structures. **c)** Simulated reflection-loss curves of the planar slab Co- and FO-based structures. **d)** Simulated reflection-loss curves of the pyramid Co- and FO-based structures.

Table S1. Finite-band absorption and structural utilization metrics of different reference structures.

| Structure | Material | Thickness | f_low | EAB | Average RL | I_B_ | Coverage ratio | Structural utilization score |
| --- | --- | --- | --- | --- | --- | --- | --- | --- |
| Slab-Co | Co | 11 mm | 2 | 2.18 | -6.27 dB | 1.394 | 5.74% | 0.034% |
| Slab-FO | FO | 11 mm | 2.46 | 2.46 | -6.72 dB | 1.540 | 6.49% | 0.043% |
| SL-Co | Co | 11 mm | 2.72 | 22.53 | -11.27 dB | 1.981 | 59.46% | 25.94% |
| SL-FO | FO | 11 mm | 3.44 | 19.36 | -11.21 dB | 1.902 | 50.96% | 17.13% |
| Pyramid-Co | Co | 11 mm | 7.55 | 32.45 | -20.61 dB | 2.133 | 85.41% | 72.01% |
| Pyramid-FO | FO | 11 mm | 3.39 | 31.22 | -19.31 dB | 2.124 | 83.66% | 64.4% |
| DL | Co/FO | 11 mm | 2 | 38 | -15.72 dB | 2.187 | 100% | 82% |

**Note:** The Slab-Co and Slab-FO samples in this table refer to simple planar material slabs calculated using the same finite-band structural utilization formula as other reference structures. They should be distinguished from the separately defined optimized planar reference used only as a baseline in the theoretical comparison.

Calculation note: The effective absorption bandwidth (EAB) was defined as the accumulated frequency range satisfying RL <= -10 dB within 2-40 GHz. The coverage ratio was calculated as EAB/(40-2). The finite-band descriptor I_B_ was calculated from the capped integral of ln(1/|S11|) over the same target band, where the cap prevents isolated extremely deep absorption valleys from dominating the metric. The structural utilization score was then calculated within this fixed-thickness and fixed-reference framework; it should not be interpreted as the efficiency relative to a universal fundamental limit.

# 5 NN-GA settings

Table S2. Design variables and bounds used in the NN-GA optimization.

| Category | Parameter | Symbol | Candidate values / range | Unit | Physical role |
| --- | --- | --- | --- | --- | --- |
| Geometry | Outer characteristic radius / size | r | 7, 9, 11, 13 | mm | Controls the main propagation path and low-frequency resonance scale of the loop structure. |
| Geometry | Outer loop / diameter parameter | d1 | 1.5, 2.0, 2.5 | mm | Regulates the local coupling gap and impedance transition in the nested structure. |
| Geometry | Inner loop/ diameter parameter | d2 | 1.5, 2.0, 2.5 | mm | Adjusts the secondary coupling path and modifies the broadband resonance distribution. |
| Thickness | Structural height | h | 5, 7, 9, 11, 13 | mm | Determines the overall absorber thickness and the available propagation/storage length. |

**Note:** A full-factorial CST parameter sweep was first performed using the above candidate values, giving 4 × 3 × 3 × 5 = 180 initial parameter combinations. Invalid simulations were removed before neural-network training.

Terminology note: geometry refers to continuous dimensions and local shape parameters, including r1, d1, d2, and h, whereas topology refers to the connectivity, nesting relation, and organization of the electromagnetic transport and dissipation pathways.

Table S3. NN-GA optimization settings.

| Module | Parameter | Value used in the code | Description |
| --- | --- | --- | --- |
| CST simulation | Solver | Frequency-domain solver | Used for obtaining the S-parameter response of each parameter combination. |
| CST simulation | Frequency range | 2-18 GHz initial screening; final design verified over 2-40 GHz | The NN-GA code used a 2-18 GHz initial design window, and the selected structure was subsequently verified over the full 2-40 GHz band by full-wave simulation and experiment. |
| CST simulation | S-parameter | SZmax(1),Zmax(1) | Reflection response extracted from CST. |
| Performance criterion | Absorption threshold | −10 dB | Frequencies with S11 below −10 dB were counted as effective absorption bands. |
| Performance criterion | Maximum reasonable bandwidth | 16 GHz for 2-18 GHz screening | Used to suppress physically unreasonable neural-network predictions during the initial screening stage. |
| Data generation | Parameter sweep method | Full-factorial sweep | All discrete combinations of r1, d1, d2, and h were simulated. |
| Neural network | Network type | Feedforward fitting network, fitnet | Used as a surrogate model between geometric parameters and effective absorption bandwidth. |
| Neural network | Hidden layers | [10, 5] | Two hidden layers with 10 and 5 neurons, respectively. |
| Neural network | Maximum epochs | 500 | Maximum number of training iterations. |
| Neural network | Data division | 70% / 15% / 15% | Training, validation, and testing split. |
| Neural network | Input normalization | Min–max normalization | Each geometric parameter was normalized using the minimum and maximum values in the dataset. |
| Neural network | Output normalization | Min–max normalization | The effective absorption bandwidth was normalized before training. |
| GA optimization | Objective | Maximize effective absorption bandwidth | Implemented by minimizing the negative bandwidth predicted by the neural-network surrogate. |
| GA optimization | Population size | 50 | Number of individuals in each generation. |
| GA optimization | Maximum generations | 100 | Maximum number of GA iterations. |
| GA optimization | Elite count | 3 | Number of best individuals retained in each generation. |
| GA optimization | Crossover fraction | 0.8 | Fraction of the next generation produced by crossover. |
| GA optimization | Mutation function | Adaptive feasible mutation | MATLAB mutationadaptfeasible was used. |
| GA optimization | Function tolerance | 1 × 10⁻⁶ | Stopping tolerance for the objective function. |
| GA optimization | Initial population | Best scanned design + Latin hypercube samples | The best CST-scanned structure was included in the initial population, and the remaining individuals were generated using Latin hypercube sampling. |
| Final validation | Validation method | CST re-simulation | The optimized parameters predicted by GA were finally verified by full-wave CST simulation. |

# 6 Comparison with other structures

Table S4. Performance benchmark comparison with representative ultrabroadband microwave absorbing structures.

| Ref. | Absorbing structure | Thickness | Absorption band / GHz | EAB / GHz | Fabrication method | Wide-angle / conformal capability | RCS validation |
| --- | --- | --- | --- | --- | --- | --- | --- |
| [B1] This work | Bioinspired nested dual-loop architecture | 11 mm | 2–40 | 38 | Dual-material 3D printing | Curved-target conformal validation | Missile-scale curved target; equivalent RCS reduced to 1% of metal |
| [B2] Wang et al. | Multi-layer nested square-frame metastructure with random deflection | 9.6 mm | 2.2–40 exp.; 4.1–40 sim. | 37.8 exp. | FDM 3D printing | Wide-angle absorption up to 60° | Planar wide-angle RCS/reflection evaluation |
| [B3] Qu et al. | Gradient honeycomb-perforated 3D metamaterial | 9.3 mm | 2–40 | 38 | Composite fabrication + structural assembly | Stable to 75° TE/TM | Planar RCS simulation |
| [B4] Liu et al. | Curved-wall composite honeycomb structure | NR | 2–40 | 38 | Composite forming / structural assembly | Angle stability within 40° | Structural stealth-related validation |
| [B5] Sun et al. | Functionally gradient octet-truss metastructure | 15 mm | 2.84–40 | 37.16 | 3D printing | Stable to 60° TE/TM | UAV-related RCS reduction |
| [B6] Wang et al. | Large-scale SiC honeycomb metastructure | NR | 4.85–39.49 | 34.64 | Material-extrusion ceramic 3D printing | Oblique incidence, high-temperature stability | Not mainly curved-target RCS |
| [B7] Liu et al. | 3D radar absorption composite hierarchical grid structure | NR | 2.6–4 and 6.6–40 | 34.8 | Molding + interlocking | Structural load-bearing | Radar stealth composite structure |
| [B8] Wang et al. | HCMM-based flexible metamaterial absorber | 5.0 mm | 3.7–40 | 36.3 | Multiscale material synthesis + macroscopic metasurface | Flexible absorber | NR |
| [B9] Jiang et al. | 3D printed honeycomb metamaterial absorber | ~15.5 mm | 3.53–24.00 | 20.47 | 3D printing + screen printing | TM absorption maintained to 70° | NR |
| [B10] Liu et al. | Prism–honeycomb nested structure | 7 mm | 1–18 exp.; 3.08–18 sim. | 17.0 exp. | Dual-material FDM 3D printing | Wide-angle absorption | RCS reduction ≈ 5 dB vs PEC |

**Note:**

**[B1]** This work. The present manuscript reports 2–40 GHz absorption at 11 mm, structural efficiency from 63.46% to 82%, and curved-target RCS reduction to 1% of the metallic counterpart.

**[B2]** Wang, L.; Chen, Z.; Zhuo, S.; Zhang, Y.; Ma, T.; Chen, M. *Thin-thickness 3D-printed dielectric loss multi-layer nested ultra broadband radar absorbing metastructure with wide incident angle*. **Composites Science and Technology** 2026, 281, 111633. The abstract reports 2–40 GHz at 9.6 mm, while the detailed result section gives 4.1–40 GHz for simulation and 2.2–40 GHz for experiment.

**[B3]** Qu, N.; Sun, H.; Sun, Y.; He, M.; Xing, R.; Gu, J.; Kong, J. *2D/2D coupled MOF/Fe composite metamaterials enable robust ultra-broadband microwave absorption*. **Nature Communications** 2024, 15, 5642. This work reports 2–40 GHz absorption at 9.3 mm and stable TE/TM oblique-incidence performance within 75°.

**[B4]** Liu, Z.; Zhang, R.; Wang, S.; Zhao, W.; Yu, G.; Wu, L. *Design and fabrication of an all-composite ultra-broadband absorbing structure with superior load-bearing capacity*. **Composites Science and Technology** 2023, 240, 110094. It reports 2–40 GHz absorption, average reflectivity of −16.5 dB, density of 0.21 g cm⁻³, and strength of 31.3 MPa.

**[B5]** Sun, C.; Li, D.; Liu, T.; Liao, W. *Design of functionally gradient metastructure with ultra-broadband and strong absorption*. **Composites Part B: Engineering** 2024. It reports −10 dB absorption from 2.84 to 40.0 GHz and −15 dB absorption from 8.51 to 40.0 GHz, with TE/TM stability up to 60°.

**[B6]** Wang, W.; Li, Z.; Gao, X.; Huang, Y.; He, R. *Material extrusion 3D printing of large-scale SiC honeycomb metastructure for ultra-broadband and high temperature electromagnetic wave absorption*. **Additive Manufacturing** 2024, 85, 104158. It reports 4.85–39.49 GHz effective bandwidth and high-temperature stability for a SiC honeycomb metastructure.

**[B7]** Liu, Z.; Wang, S.; Shao, J.; Wu, L. *3D radar stealth composite hierarchical grid structure with extremely broadband absorption performance and effective load bearing*. **Composites Part B: Engineering** 2022. It reports 34.8 GHz total −10 dB bandwidth over 2.6–4 GHz and 6.6–40 GHz with average reflectivity of −17.4 dB.

**[B8]** Wang, J.; Wu, Z.; Xing, Y.; Liu, L. *Multi-Scale Design of Ultra-Broadband Microwave Metamaterial Absorber Based on Hollow Carbon/MXene/Mo₂C Microtube*. **Small** 2023. It reports flexible HCMM-based MMA absorption from 3.7 to 40.0 GHz at 5.0 mm thickness.

**[B9]** Jiang, W.; Yan, L.; Ma, H.; Fan, Y.; Wang, J.; Feng, M.; Qu, S. *Electromagnetic wave absorption and compressive behavior of a three-dimensional metamaterial absorber based on 3D printed honeycomb*. **Scientific Reports** 2018, 8, 4817. It reports >90% absorption from 3.53 to 24.00 GHz and TM oblique-incidence robustness up to 70°.

**[B10]** Liu, S.; Zhou, Y.; Zhang, F.; Wang, Q.; Zhao, X.; Chao, B.; Wu, H. *A Novel Full-Band Microwave Absorber Based on Scattering Enhanced Prism-Honeycomb Nested Structure*. This work reports 1–18 GHz experimental absorption at 7 mm and ≈5 dB RCS reduction relative to PEC.

# 7 Field energy supplement

Figure S7–S14 constitute the full supplementary field-energy dataset corresponding to the mechanism analysis in the main text. Specifically, Figure S7 and Figure S8present the electric-field distributions of the dual-loop and single-loop structures, Figure S9 and Figure S10 show the corresponding magnetic-field distributions, Figures Figure S11 and Figure S12 provide the energy-loss maps, and Figures Figure S13 and Figure S14 give the power-flow distributions over the representative frequency range from 4 to 36 GHz. Here, z1 and z2 denote two mutually orthogonal polarization directions of the electromagnetic field. The complete field-energy behavior of the structure should therefore be understood as the superposition of the z1 and z2 responses rather than from either polarization alone.

From low to high frequency, the field-energy evolution exhibits a clear topology-dependent trend. In the low-frequency regime, the dual-loop structure already shows stronger inter-ring electric coupling and more connected magnetic activity than the single-loop case, indicating earlier activation of collective resonance pathways. In the intermediate-frequency regime, the dual-loop configuration develops more persistent magnetic confinement and deeper power penetration, suggesting that the nested topology is more effective at injecting and storing electromagnetic energy within the structure. In the high-frequency regime, the most pronounced difference appears in the energy-loss and power-flow distributions: the dual-loop structure evolves toward broad, distributed multi-loss pathways with more continuous inter-cell energy transfer, whereas the single-loop structure still mainly exhibits localized edge-dominated dissipation and comparatively discrete transport channels. Overall, the dual-loop topology transforms isolated local resonances into a more connected broadband dissipation network, which is fully consistent with the main-text interpretation of enhanced coupling efficiency, increased storage gain, and stronger bandwidth expansion.

The representative maps at 4, 16, and 32 GHz further illustrate a continuous frequency-dependent evolution of the coupled modes. At 4 GHz, the inner and outer loops respond collectively, extending the effective propagation path and supporting magnetic-field confinement. At 16 GHz, stronger inter-ring interaction and overlapping loop modes produce broader field localization and deeper power penetration. At 32 GHz, higher-order edge and interfacial modes become more prominent and generate spatially distributed loss pathways. These regimes are not separated by abrupt boundaries; together they form a continuously evolving modal network that sustains the 2-40 GHz response.

**Figure S7** Electric field distribution of dual-loop structure.

**Figure S8** Electric field distribution of single-loop structure.

**Figure S9** Magnetic field distribution of dual-loop structure.

**Figure S10** Magnetic field distribution of single-loop structure.

**Figure S11** Energy loss of dual-loop structure.

**Figure S12** Energy loss of single-loop structure.

**Figure S13** Power flow of dual-loop structure.

**Figure S14** Power flow of single-loop structure

# 8 Method supplement

Table S5. Composition of composite materials.

| Name | Content (wt%) | | | |
| --- | --- | --- | --- | --- |
|  | Co | Fe_3_O_4_ | PLA | TPU |
| Co10 | 10 | 0 | 81 | 9 |
| Co30 | 30 | 0 | 63 | 7 |
| C050 | 50 | 0 | 45 | 5 |
| Co70 | 70 | 0 | 27 | 3 |
| FO10 | 0 | 10 | 81 | 9 |
| FO30 | 0 | 30 | 63 | 7 |
| FO50 | 0 | 50 | 45 | 5 |
| FO70 | 0 | 70 | 27 | 3 |
